# Supplementary material for: The Labour Conditions and Health of Migrant Agricultural Workers in Spain: A Qualitative Study
Source: Healthcare (Basel). 2025 Jul 31;13(15):1877. doi: 10.3390/healthcare13151877 (PMC12345661; doi:10.3390/healthcare13151877)
Supplement: Supplementary file 1 [file healthcare-13-01877-s001.zip › healthcare-3734786-supplementary.pdf]

Supplementary Materials. Interview Guide for Professionals assisting Migrant Agricultural Workers

Sociodemographic Data

- Age
- Sex
- Education
- Profession
- Place of residence/work
- Years of experience

Migration History

- What is the general profile of seasonal agricultural workers? (men/women, regions of origin, young adults/adults, administrative status…)
- Have these profiles changed over time?
- What differences exist between workers who reside permanently in Spain and move between Autonomous Communities, and those who travel only during the agricultural season and return to their country afterwards (e.g., hired-at-origin workers)?
- Could you describe the usual trajectory that these people follow before arriving to work in this region? Please specify places and timing if possible
- What are the main difficulties migrants face in the host country?

Living Conditions

- Where do the seasonal workers in this region live and in what conditions?
- What are the characteristics of the settlements/substandard housing in the region?
- What differences exist between the housing conditions of workers who work permanently in a region and those who move from region to region?
- Explore health-related aspects of settlements/substandard housing:
  - Access to drinking water
  - Access to other basic services such as electricity or heating and basic appliances such as refrigerators or washing machines
  - Waste disposal (both garbage and human waste)
  - Food management (storage, cooking, etc.)
  - Environmental protection (insulation against extreme temperatures, rain, or snow, etc.)

Health Acccess Block

- How did COVID-19 impacted this population?
- What was your role/experience in managing the second wave of COVID-19 in your region with migrant seasonal workers?

- Based on your experience, what are the main health problems seasonal agricultural workers suffer?
- How do you think working and living conditions affect their health?
- Explore measures taken to seek healthcare, type of care received, response provided, follow-up, and evaluation of available resources
  - Barriers or factors that hinder access and care
  - Factors that facilitate care
  - Influence of administrative status on the use of services (healthcare, social services…)
  - Health card management
  - Suggestions for improving healthcare services for migrants

Closing Questions

- What are the main difficulties faced by professionals who support seasonal workers? What are your needs?
- Finally, is there anything else that has not been addressed but you consider important to add?
